# Brain Metastases From HER2 Breast Cancer That Achieved Complete Response With Trastuzumab Deruxtecan Without Any Local Treatment

**DOI:** 10.7759/cureus.85011

**Published:** 2025-05-29

**Authors:** Katsuhiro Yoshikawa

**Affiliations:** 1 Breast Surgery, Takatsuki General Hospital, Takatsuki, JPN

**Keywords:** advanced breast cancer, brain metastasis, breast cancer, her2 positive, systemic therapy, t-dxd, trastuzumab deruxtecan

## Abstract

The blood-brain barrier (BBB) prevents high molecular weight drugs from reaching the brain, and only some low molecular weight drugs have been effective against brain metastases (BMs) from breast cancer. Therefore, local therapy, such as surgery or radiotherapy, has been the first choice and the standard treatment for BMs. However, trastuzumab deruxtecan (T-DXd), which has recently been introduced, demonstrates a high penetration rate into BM lesions and is believed to be highly effective. We herein present a case of complete response to T-DXd alone, without local therapy, in a patient with BMs from human epidermal growth factor receptor 2 (HER2)-positive breast cancer. The patient was a 56-year-old premenopausal woman with estrogen receptor-positive, progesterone receptor-positive, HER2(+) left breast cancer; left axillary, left cervical, and left submandibular lymph node metastases; and multiple BMs. Lymphedema of the left upper limb was observed. However, no cerebral neurological symptoms were noted. She refused local therapy for BMs because her job required advanced calculations, and she could not afford the possibility of cognitive decline. She chose trastuzumab + pertuzumab + docetaxel (TPD) triweekly as the first-line therapy, and at the end of six cycles, the BMs were stable and the metastatic lesions in the body showed a complete response; however, peripheral sensory neuropathy due to docetaxel appeared. Therefore, docetaxel was discontinued, and tamoxifen (TAM) was started instead. Trastuzumab + pertuzumab (TP) was continued. At the end of six cycles of TP-TAM (a total of 12 cycles of TP), because BM appeared to be progressive on imaging tests, TP-TAM was discontinued, and T-DXd was introduced; however, no neurological symptoms were observed. At the end of the six cycles, BMs had disappeared. Although local therapy is the standard for BMs, the National Comprehensive Cancer Network guidelines state that systemic therapy can be prioritized before local therapy in the absence of active neurological symptoms. T-DXd is an antibody-drug conjugate that penetrates the BBB disrupted by metastasis and is thought to exert its effectiveness by penetrating the lesion through a bystander effect. Furthermore, it is thought to be a safe treatment option for patients with unacceptable complications of local therapy, as in our case.

## Introduction

The prognosis of human epidermal growth factor receptor 2 (HER2)-positive breast cancer has improved dramatically with the advent of HER2-targeted therapy; however, the prognosis for patients with brain metastases (BMs) remains poor. Approximately 30%-50% of patients with HER2-positive advanced breast cancer develop BMs [1]. Unfortunately, this subgroup of patients suffers from poor quality of life and short life expectancy, with overall survival (OS) ranging between two and 16 months [2].

The blood-brain barrier (BBB), a highly selective diffusion barrier in the brain, prevents most drugs - such as antibodies and antibody-drug conjugates (ADCs) - from reaching the brain, rendering them less effective. Only some small-molecule drugs have shown limited efficacy against BMs in breast cancer [3,4]. Therefore, local treatments such as surgery and radiotherapy have been the first choice for BMs, with systemic therapy considered an adjunct to local therapy. Consequently, the prognosis is poor for patients with BMs who cannot be treated locally for various reasons.

However, trastuzumab deruxtecan (T-DXd), an HER2-directed ADC carrying a deruxtecan-derivative topoisomerase I inhibitor payload, has been suggested to have the potential to reach BMs and be highly effective [3,4]. Here, we report a case of BMs from HER2 breast cancer that achieved a complete response on magnetic resonance imaging (MRI) with T-DXd, without any local treatment.

## Case presentation

**Figure 1:**
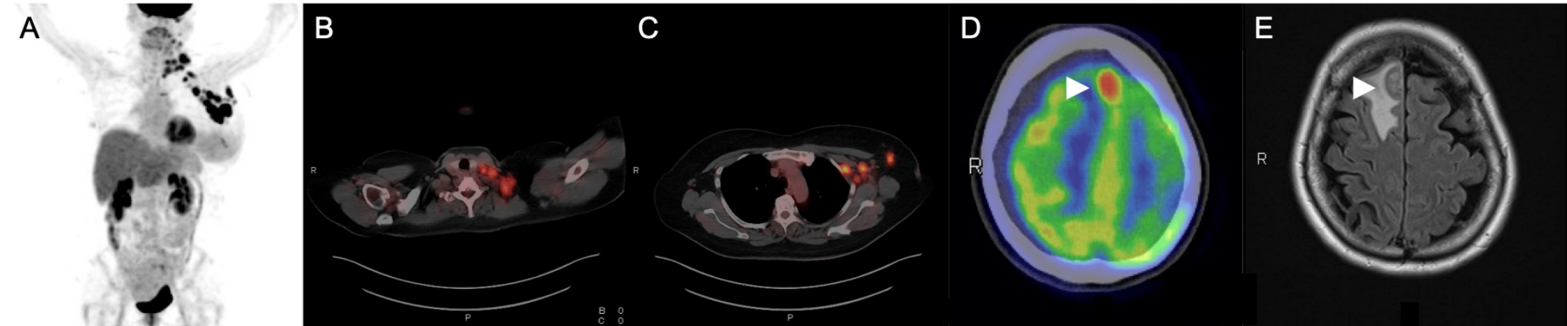
Imaging findings before treatment initiation PET-CT scan (A, B, C, D) and MRI of the brain (E). The arrowhead points to the largest of the multiple brain metastases. PET-CT: Positron emission tomography/computed tomography; MRI: Magnetic resonance imaging

A 56-year-old woman presented with edema of the left upper limb in April 2023, a multiple mass of approximately 1 cm each in left breast, and thickening and redness of the associated skin and metastatic axillary lymph nodes, cervical lymph node, and submandibular lymph node, as observed on positron emission tomography/computed tomography (PET-CT) (Figures 1A-1C).

MRI revealed multiple brain masses. The largest mass, which measured 2.0 cm × 1.5 cm and had extensive surrounding edema, was located in the right frontotemporal lesion (Figures 1D-1E). The diagnosis was established through a core needle biopsy of the left breast, which revealed estrogen receptor (ER)-positive, progesterone receptor-positive, HER2-positive invasive breast carcinoma of no special type. Based on the imaging and histological examination results, the patient was diagnosed with hormone receptor-positive, HER2-positive metastatic breast cancer.

She had no neurological symptoms, and her Karnofsky performance status score was 100%. Because her profession required complex calculations and she did not want to suffer from neurocognitive decline, she preferred to be treated with systemic therapy alone, without local treatment.

**Figure 2:**
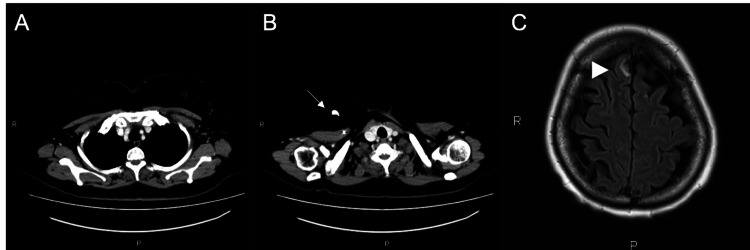
Imaging findings after six cycles of TPD CT scan (A, B) and MRI of the brain (C). The white arrow points to the central venous port. The arrowhead points to the largest of the multiple brain metastases. TPD: Trastuzumab + pertuzumab + docetaxel; CT: Computed tomography; MRI: Magnetic resonance imaging

Trastuzumab 6 mg/kg + pertuzumab 420 mg + docetaxel 75 mg/m^2^ (TPD) triweekly was started first, as TPD is considered the first choice of treatment for HER2-positive advanced breast cancer [1], and trastuzumab + pertuzumab (TP) has been reported to reach the brain despite the presence of the BBB [5,6]. After one treatment cycle, the edema of the upper limb disappeared. Chest and abdominal computed tomography (CT) and brain MRI were performed at the end of six cycles. The peritumoral edema showed improvement; however, the changes in BM size remained consistent with stable disease (SD), whereas the metastatic lesions in the body and neck exhibited complete response (CR) (Figures 2A-2C).

**Figure 3:**
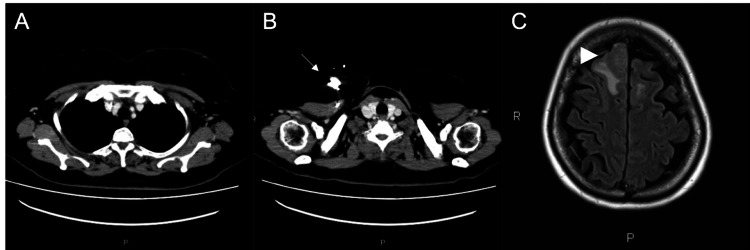
Imaging findings after six cycles of TP-TAM CT scan (A, B) and MRI of the brain (C). The white arrow points to the central venous port. The arrowhead points to the largest of the multiple brain metastases. TP-TAM: Trastuzumab + pertuzumab + tamoxifen; CT: Computed tomography; MRI: Magnetic resonance imaging

Because of good disease control, TP was continued; however, docetaxel was discontinued because of peripheral sensory neuropathy, and tamoxifen 20 mg (TAM) was started instead. The same imaging tests as before were performed at the end of six cycles, revealing that the body and neck lesions continued to show CR; however, the brain lesions demonstrated progressive disease (Figures 3A-3C).

**Figure 4:**
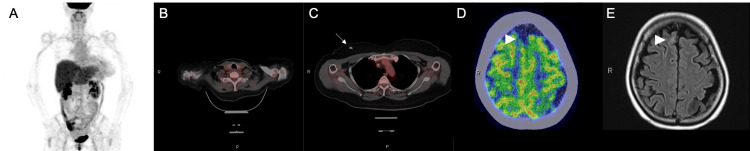
Imaging findings after six cycles of T-DXd PET-CT scan (A, B, C, D) and MRI of the brain (E). The white arrow points to the central venous port. The arrowhead points to the largest of the multiple brain metastases. T-DXd: Trastuzumab deruxtecan; PET-CT: Positron emission tomography/computed tomography; MRI: Magnetic resonance imaging

TP-TAM was discontinued, and T-DXd (5.4 mg/kg) was initiated as the second-line treatment. Although tucatinib has also been suggested to be effective for BMs, it was not used because it was not available in Japan at the time of the patient's treatment selection. Imaging tests were performed after six cycles. The results indicated that all lesions, including those in the brain, achieved CR (Figures 4A-4D).

Currently, the patient has received >20 cycles of T-DXd and has been living for two years with a stable disease condition while maintaining a good quality of life.

## Discussion

BMs develop in approximately 15% of patients with advanced breast cancer and are prevalent in the HER2 type at 30%-50% compared with other subtypes [1]. The median OS of metastatic HER2 breast cancer is approximately 58 months [7]; however, when BMs develop, the prognosis is 13-19 months, which is poor [8]. BM lesions are protected by the BBB, so high-molecular-weight drugs cannot penetrate them and are considered less effective [9]. Therefore, local therapies such as surgery and radiotherapy have generally been prioritized for BMs, whereas systemic therapy with drugs has been considered an adjunctive therapy. In cases with few metastases, surgical resection or stereotactic radiosurgery is chosen, whereas whole-brain radiation is selected for cases with numerous metastases [10]. In addition, combining these therapies has been attempted. By repeating these treatments each time the BMs progress, the metastatic lesions can be controlled for a certain period [11]. However, all these local therapies can also damage normal brain cells, leading to cognitive decline [12]. This can be a serious complication, particularly in young patients, because it affects their ability to work.

Systemic therapy for BMs may be effective when the BBB is compromised by the tumor; the response rates for TP and trastuzumab emtansine (T-DM1) for BMs in HER2 breast cancer have been reported to be 11% and 21%, respectively [6,13,14]. Some case reports of responses to endocrine therapy for BMs in ER-positive HER2-negative breast cancer suggest that endocrine therapy may be effective in treating BMs; however, prospective studies are needed to confirm the results [15]. Lapatinib, a small-molecular drug, has a good intracranial response rate of 65% [14,16] but is inferior to other drugs affecting the whole body at 22% (trastuzumab and T-DM1: 80% and 44%, respectively) [14,17,18]. They were not the first choice for BM treatment when compared with local therapy, and drug therapy was used either as maintenance therapy after local therapy or as an alternate plan when patients did not want to lower their cognitive function. However, newer anti-HER2 drugs such as T-DXd and tucatinib have been reported to penetrate BMs and exert their effects. In particular, T-DXd is thought to attack BMs through a bystander effect after passing through the BBB [4]. The response rates of T-DXd and tucatinib to BMs were 64% and 47%, respectively, and those to whole metastases were 80% and 41%, respectively, making them very effective compared with conventional drugs [14,19,20]. Based on the results, as of 2024, the National Comprehensive Cancer Network (NCCN) guidelines state that systemic therapy with T-DXd or tucatinib may be administered for HER2 breast cancer BMs that do not require urgent local therapy [14]. Although the NCCN guidelines limit the use of T-DXd to inactive BMs, the results of the TUXEDO-1 [4] and ROSET-BM [3] trials and other studies have demonstrated its effectiveness for treating BMs, and our reports have shown high tumor reduction and systemic maintenance of CR, including intracranial lesions.

## Conclusions

We presented a case of metastatic breast cancer in a woman with multiple BMs that was successfully treated with systemic therapy. The patient achieved a CR and long-term survival with systemic drug therapy using T-DXd, without any local therapy for the BMs. This may be a very effective treatment modality for cases where local therapy is not available. T-DXd requires monitoring for adverse events such as interstitial lung disease; however, it could be an effective option for patients with systemic metastases, including BMs, provided that interstitial lung disease is effectively managed. Nevertheless, this is only one case, and prospective studies will be necessary to change the standard therapy in the future.
